# Knowledge, perceptions and practice of cervical cancer prevention among female public secondary school teachers in Mushin local government area of Lagos State, Nigeria

**DOI:** 10.11604/pamj.2017.28.221.13980

**Published:** 2017-11-10

**Authors:** Mariam Adeola Toye, Kehinde Sharafadeen Okunade, Alero Ann Roberts, Omolola Salako, Ezekiel Sofela Oridota, Adebayo Temitayo Onajole

**Affiliations:** 1 Department of Medicine and Surgery, College of Medicine, University of Lagos, Lagos, Nigeria; 2 Department of Obstetrics and Gynaecology, College of Medicine, University of Lagos, Lagos, Nigeria; 3 Department of Community Health, College of Medicine, University of Lagos, Lagos, Nigeria; 4 Cancer Information Service, Sebeccly Cancer Care & Support Center, Lagos, Nigeria

**Keywords:** Cervical cancer, HPV vaccine, Lagos, Mushin, prevention, Papanicolaou

## Abstract

**Introduction:**

Cervical cancer is the most common gynecological cancer and a leading cause of cancer death in women in Nigeria. This study was aimed to assess the knowledge, perception, and practice of cervical cancer prevention among female public secondary school teachers in Mushin, Lagos.

**Methods:**

This was a cross-sectional study carried out among female secondary school teachers in Mushin, Lagos. The participants were selected by a two-stage random sampling method and relevant data were collected with the use a self-administered questionnaire. Data entry and analysis were done using Epi-info version 7.2 statistical software and descriptive statistics were computed for all data.

**Results:**

The knowledge of cervical cancer and its prevention was 100.0% among the respondents. The most commonly known method of cervical cancer screening identified by the respondents was Papanicolaou smear (91.4%). More than half of the women (67.0%) have had at least one cervical cancer screening done previously. Only 2.2% of the respondents have had HPV vaccine given to their female teenage children in the past despite the acceptance rate for HPV vaccination being 76.2%.

**Conclusion:**

This study, unlike most previous studies in other regions of Nigeria and most part of sub-Saharan Africa, has demonstrated a relatively high level of awareness about cervical cancer, its cause, risk factors and prevention. However, conversely, the absence of a national health programme means that screening and vaccination centers are not available, accessible or affordable.

## Introduction

Cervical cancer is the most common gynecological cancer and a leading cause of cancer death in women in Nigeria [1]. It ranks third among the most common cancers affecting women the world over with an estimated 529,000 new cases in 2008, 85% of which were recorded in the developing world [2, 3]. Cervical cancer contributes to 20 to 25% of all cancers among women in sub-Saharan Africa, about twice the percentage in women worldwide. In spite of the fact that cervical cancer is preventable, the incidence is expected to increase to almost double the current rate by 2025 [3]. Cervical cancer continues to cause the deaths of almost 270,000 women worldwide each year. About 87% of these occurred in developing countries, particularly in the rural areas. Its incidence in the Sub-Saharan African countries ranges from 30 to 40 per 100,000women [3, 4]. In Nigeria, cervical cancer kills one woman every hour and over 9000 women every year [2]. More than three-fourths of cervical cancer patients are diagnosed at advanced stages leading to poor prospects of long-term survival and cure. This is due to the non-existence of a national screening programme, lack of infrastructure, poorly trained health staff and huge financial cost. It evidently constitutes a huge public health burden as the attendant loss of lives is needless due to its preventable nature [5]. Screening is currently viewed as the most effective approach for cervical cancer control and it is associated with reduced incidence and mortality from the disease [6]. The primary causative agent is the human papillomavirus (HPV) with the high-risk genotypes (HR-HPV) being responsible for the development of invasive cancer. Over 70% of all cases of cervical cancer are directly due to infection with HPV-16 and 18 strains thus making it the only human cancer whose necessary cause is known [7-9].

This knowledge forms part of the basis for designing successful preventive strategies. Risk factors for HPV infection and subsequent cervical cancer include early age of first sexual exposure, multiple sexual partners, smoking and immunosuppression [10]. Although the mean age of diagnosis is 50 years, women as young as 17 years can also develop the disease with the highest risk group being 25-49 years [4]. Conventionally, Pap smear, combined with treatment of cervical pre-cancerous lesions and early-stage cancer, has been successful in preventing up to 80% of invasive cervical cancer cases in the developed world [11, 12]. In developing countries, however, high rates of cervical cancer mortality still persist due to lack of effective screening programs and low uptake of Pap smear testing [13]. Newer technologies such as the HPV/DNA test (QIAGEN, Gaithersburg, MD, USA), cervicovaginal self-sampling, and HPV vaccination have the potential to increase screening and reduce cervical cancer in developing countries [14]. However, awareness about new screening and cervical cancer prevention methods remains low among most women, with studies recording almost no awareness of HPV infection, HPV screening and adolescent vaccination for the prevention of future disease [15]. This study is therefore aimed to determine the level of knowledge, perception, and practice of cervical cancer prevention among female public secondary school teachers in Mushin Local Government Area of Lagos state as this may have far-reaching effects because teachers are most often well respected and are viewed as being very knowledgeable by people in their communities. It is thus hoped that the result of this study would help to increase awareness and suggest appropriate interventions to foster improved screening among women and also encourage parents to vaccinate their daughters against cervical cancer.

## Methods

Mushin Local Government Area is located in Lagos, Southwestern Nigeria. It is situated 10km North of the Lagos city core and is one of the nation's 774 local government areas. It lies on the railway from Lagos and at the intersection of roads from Lagos, Shomolu, and Ikeja (the Lagos State capital). It is bounded by Oshodi-Isolo Local Government in the north, Shomolu Local Government in the east and Mainland Local Government in the south [16]. For most part, it is an overcrowded residential area with inadequate sanitation and low-quality housing. This was as a result of uncontrolled continuing expansion from 1950. The population size as at the 2006 census was 633,009. There is a large industrial estate in the area and commercial activities include spinning and weaving cotton, shoe manufacturing, bicycle and motorized-cycle assembly and the production of powdered milk. Agricultural produce is brought for sale in the large markets of Ojuwoye, Daleko and Ladipo [17]. There were a total of 15 public secondary schools in the area with each having junior and senior divisions. The number of secondary school teachers is estimated to be 1080 with a female to male ratio of about 3:2 [17]. This was a cross-sectional survey involving female public secondary school teachers in Mushin Local Government Area. The number of female teachers at the time of study is 648, with an average of 43 in each secondary school. The minimum sample size (178) for the study was calculated using the statistical formula developed by Fisher [18] and another 10% of the minimum sample size was added to make up for attrition and non-response. The final sample size used for the study was therefore 200 when approximated to the nearest whole number. A simple random technique by balloting was used to select 8 schools and 25 consenting female teachers in each of the selected school were consecutively recruited for the study. Relevant data were collected with the use of pre-tested self-administered questionnaire. A well-structured, 27-item validated questionnaire was adapted for the study [19] to collect information on the bio-data, socioeconomic status of the participants, participants' awareness, perceptions and practice of cervical cancer prevention. Data entry and analysis were done Epi-info version 7.2 statistical software manufactured by the US Centres for Disease Control and Prevention. The quantitative data were presented in tables and charts and analyzed as descriptive frequencies, percentages and cross tabulations. Ethical approval for the study (HREC Number: ADM/DCST/HREC/2152) was obtained from the Health Research and Ethics Committee of the Lagos University Teaching Hospital prior to the commencement of the study and the ethical principles according to the Helsinki declaration were considered during the course of the research. Permission was also sought from the Local Education District in charge of Mushin Local Government and each school's principal. Important ethical principles considered during the study were as follows: informed written consents were taken from the participants prior to their enrolment; the investigators ensured strict confidentiality of all participants' information; all the participants also stand to benefit from the policy that may eventually emanate from the findings of this study.

## Results

Out of 200 self-administered questionnaires, 185 were correctly filled and analyzed for this study thus giving a response rate of 92.5%. Table 1 showed that the age of respondents in the study ranged from 27 to 62 years with a mean age of 42.6 ± 3.2 years. More than half (56.8%) of the respondents were from the Yoruba ethnic group with the same proportion also being of the Christian faith. A vast majority of the respondents (90.8%) were married with almost two-third (63.8%) of them having at least 3 children (mean parity = 3.35 ± 1.44). As shown in Table 2, all of the respondents (100.0%) have heard of cervical cancer with the common sources of information being from the medical personnel (75.7%), media (40.0%), family members (40.0%), internet (44.3%) and friends (43.2%). There was a generally variable level of awareness of the risk factors for cervical cancer among the respondents with the correctly identified factors being multiple sexual partners (61.1%), family history of cervical cancer (60.0%) and HPV infection (43.8%). All of the respondents were aware of at least one method of cervical cancer prevention (Table 3) with the commonest sources of information being from the print and electronic media (71.9%) and medical personnel (64.3%). The most commonly known method of cervical cancer prevention among the respondents was Papanicolaou smear (91.4%) while the least known methods were liquid-based cytology and visual inspection with Lugols iodine (4.3% each). In Table 4, majority of the women surveyed (95.7%) agreed that cervical cancer can be prevented. A large proportion (96.2%) also agreed that awareness of risk factors and healthy lifestyle can prevent cervical cancer. A vast majority of respondents (87.6%) agreed that women should be screened at least once in their lifetime while up to 76.2% of respondents favour vaccination of their teenage girls with the HPV Vaccine. Regarding the practice of cervical cancer prevention among respondents as revealed in Table 5, more than half of the women (67.0%) have had at least one cervical cancer screening done previously. Among those respondents who had never been screened, the most common reason adduced for this was that they never thought it was necessary (49.2%). Only 2.2% of the respondents have had HPV vaccine given to their female teenage children in the past and this was despite the acceptance rate of HPV vaccination being 76.2% among the respondents. The vast majority of the women (54.1%) who had never had any of their children vaccinated attributed this to lack of awareness of the existence of the vaccine and its benefits.

**Table 1:** Socio-demographic characteristics of the respondents (n = 185)

Characteristics	Frequency	%
**Age**		
25-29	9	4.9
30-34	26	14.1
35-39	31	16.8
40-44	41	22.2
45-49	31	16.8
50-54	40	21.6
≥55	7	3.8
Age range = 27-62 years	**Mean age ± SD = 42.6 ± 3.2 years**	
**Marital status**		
Single	9	4.9
Married	168	90.8
Divorced	4	2.2
Widowed	4	2.2
**Parity**		
0	4	2.2
1	16	8.6
2	47	25.4
3	67	36.2
4	36	19.5
≥5	15	8.1
Mean parity ± SD = 3.35 ± 1.44		
**Ethnic group**		
Yoruba	105	56.8
Igbo	34	18.4
Hausa	4	2.2
Others	42	22.7
Religion		
Christianity	105	56.8
Islam	75	40.5
Others	5	2.7

**Table 2:** Knowledge of cervical cancer (n = 185)

Knowledge	Frequency	%
**Ever heard of cervical cancer**		
Yes	185	100.0
No	0	0.0
**Source(s) of information about cervical cancer**		
Family	74	40.0
Friends & Colleagues	80	43.2
Media (newspaper/radio/television)	74	40.0
Internet search engine	82	44.3
Social media	4	2.2
Medical personnel	140	75.7
**Awareness of the risk factors for cervical cancer**		
HPV infection	81	43.8
Having many children	72	38.9
Long-term use of oral contraceptive	20	1.1
Early exposure to sexual intercourse	65	35.1
Multiple sexual partners	113	61.1
Partners who have other sexual partners	76	41.1
Family history of cervical cancer	111	60.0
Poor hygiene	43	23.2
Low socioeconomic status	23	12.4
Type of diet	38	20.5
Smoking	113	61.1
Alcohol	13	7.0

**Table 3:** Knowledge of cervical cancer preventive methods (n = 185)

Knowledge	Frequency	%
**Awareness of cervical cancer preventive methods**		
Yes	185	100.0
No	0	0.0
**Source(s) of information about cervical cancer prevention**		
Family	36	19.5
Friends & Colleagues	48	25.9
Media (newspaper/radio/television)	133	71.9
Internet search engine	80	43.2
Social media	21	11.4
Medical personnel	119	64.3
**Knowledge of the various preventive methods**		
Papanicolaou smear	161	91.4
Liquid-based cytology	8	4.3
Visual inspection with acetic acid	59	31.9
Visual inspection with lugols iodine	8	4.3
Primary HPV screening	10	5.4
Colposcopy	25	13.5
HPV Vaccination	87	47.0

**Table 4:** Perception of respondents to cervical cancer prevention

Perception	Frequency	%
Cervical cancer can be prevented	177	95.7
Awareness of risk factors & healthy lifestyle can prevent cervical cancer	178	96.2
Screening should be done at least once in a lifetime	162	87.6
I will allow the vaccination of my girl child against HPV infection	141	76.2

**Table 5:** Practice of cervical cancer prevention among respondents

Practice	Frequency	%
Ever screened for cervical cancer (n=185)		
YES	124	67.0
NO	61	33.0
Reasons for NOT screening previously (n=61)		
Test is too expensive	4	6.6
It is too embarrassing to do the test	8	13.1
It is unnecessary	30	49.2
Test is not readily available	19	31.1
Ever had HPV vaccine given to a female teenage child (n=185)		
YES	4	2.2
NO	181	97.8
Reasons for NOT previously getting HPV vaccine for their female children (n=181)		
It is too expensive	16	8.8
Not available	13	7.2
It is unnecessary	15	8.4
Society values & stigma	8	4.4
Concerns about vaccine safety & side-effects	7	4.0
Promotes sexual promiscuity	24	13.3
Not aware of the vaccine existence and its benefits	98	54.1

## Discussion

In this study, the knowledge, perception and practice of cervical cancer prevention among female public Secondary School teachers in Mushin Local Government Area of Lagos State, Nigeria were reviewed. The participants' response rate of 92.5% noted in this study was higher than the response rates of 81.5% [20] and 88.5% [21] obtained from two other similar studies conducted in South-eastern, Nigeria. A vast majority of the respondents in this study were married (90.8%) which was not unexpected as the teachers population generally comprises of adult population of marriageable age group. The high parity reported among the respondents justified the importance of carrying out the study among this very representative group of people who will benefit the most from its findings as multiparity has been shown in many other studies to be an important risk factors for cervical cancer [22-24], and also because these women can act as the most effective custodians and vehicles for the dissemination of cervical cancer-related information not only to the community but also to their own female children. The study also showed that more than half (56.8%) of the respondents were from the Yoruba ethnic group, which is attributable to the fact that the study was done in the South Western region of the country which comprises predominantly of the Yoruba ethnic group. With an almost equal distribution of Christians and Muslims among the respondents, it showed that the findings from this study may not likely be influenced by religion unlike other similar studies carried out in South-east, Nigeria where the respondents were predominantly of the Christian faith [20, 21]. This study demonstrated a higher level of knowledge (43.8%) of the causal relationship between HPV infection and cervical cancer than the 19% knowledge level recorded in a 2008 study carried out in 3 different areas in Birmingham by Walsh and co-workers [25]. This finding was probably due to the predominantly urban and highly educated group of participants used in our study, unlike the Birmingham study which was carried out mostly in the rural communities with predominant uneducated and semi-educated respondents. In the Nigerian study done conducted by Ugwu and colleagues in Enugu state, 85.9% of the participants were aware of cervical cancer and that it was preventable [21], a finding which was slightly similar to that of this present study where the level of knowledge of cervical cancer and at least of one its preventive method is an impressive100%. Similar sources of information about cervical cancer and its preventive modalities were identified in this study just like the Enugu study [21].

The 100% level of awareness of cervical cancer in this study is a testament to the predominant highly educated and urban population of respondents recruited who would probably have been exposed to one source of information or the other. This assertion was also corroborated by the Nigerian study done in both urban and rural settings in South-eastern where the knowledge of cervical cancer screening was significantly associated with urban dwellings [20]. The study also showed that the health care personnel play a pivotal role in the dissemination of health-related information as a predominantly large proportions of the respondents learned of cervical cancer (75.7%) and its prevention (64.3%) from health care providers. This may further justify that a more personal physician-patient encounters may act as a relatively more effective source information and education than the non-personal source of information dissemination such as mass media as reported in a study carried out in Kuwait [26]. This was also corroborated by the Nigerian study where it was found that all women who had previously screened for cervical cancer were referred by their physicians [20]. However, the mass media was still reported as the most common source of information on cervical cancer and its prevention in a study by Tan et al among female University students in Malaysia [27] thus demonstrating importance also. About 95.7% of participants in our study agreed that cervical cancer can be prevented in a somewhat similar fashion to the 85.9% figure reported in Enugu, Nigeria [21]. The screening rate (67.3%) in this study was far higher than the 4.2% reported by a similar study conducted in South-east Nigeria [20]. This was understandable because of the very diverse population distribution of participants and the geographical locations used in these 2 studies. However, factors adduced by the respondents for their inability to access the available cervical cancer screening services in the two studies and other previous studies [20, 25, 26 ] were quite similar. There was a high rate of acceptance of HPV vaccination in this study (76.2%), although the figure was still less than the 88% and 95% recorded in the Birmingham and Kenyan studies respectively [25, 28] and also did not translate to the actual vaccination rate of the respondents teenage daughters which was reported at 2.2%. The reasons given for this remarkably low vaccination rate by the respondents were mainly due to the lack of sufficient information about the HPV infection and the vaccine itself and the negative sociocultural beliefs about the vaccine among the populace similarly to findings in other previous studies [29-33]. This was also supported by findings from previous UK-based studies that demonstrated generally low knowledge about HPV, but with most respondents supporting the introduction of the vaccine after provision of adequate information [34-37] and hence reinforcing the need for more educational intervention in order to raise awareness about cervical cancer and its prevention.

## Conclusion

This study, unlike most previous studies in other regions of Nigeria and most part of sub-Saharan Africa, has demonstrated a relatively high level of awareness about cervical cancer, its cause, risk factors, and prevention. However, conversely, the absence of a national health programme means that screening and vaccination centers are not available, accessible or affordable. Therefore, Sub-Saharan African region which carries the greatest burden still has the least resources to tackle this problem. Attempts to reinforcing the knowledge of cervical cancer and correcting the wrong perceptions towards cervical cancer prevention will have a positive influence on its practice and furthermore assist all stakeholders in the health sector to work together to establish a national programme for cervical cancer screening and vaccination. This will thus reverse the current dismal situation in which the only preventable cancer is still causing such a high morbidity and mortality in Nigeria and other developing countries of the world.

### What is known about this topic

- That cervical cancer is the most common gynaecological cancer and a leading cause of cancer death in women in Nigeria;
- More than three-fourths of cervical cancer patients are diagnosed at advanced stages leading to poor prospects of long-term survival and cure mainly due to the non-existence of a national screening programme, lack of infrastructure, poorly trained health staff and huge financial cost;
- Screening is currently viewed as the most effective approach for cervical cancer control and it is associated with reduced incidence and mortality from the disease but awareness about new screening and cervical cancer prevention methods remains low among most women in the developing countries.

### What this study adds

- There is a generally high level of awareness of cervical cancer, its causes, and prevention among female public secondary school teachers in Mushin Local Government area of Lagos;
- There is, however, a relatively low level of practice of cervical cancer prevention among the respondents;
- The study, therefore, revealed that further attempts should be made to reinforce the knowledge of cervical cancer and correct the wrong perceptions towards cervical cancer prevention among the Nigerian female populace.

## Competing interests

The authors declare no competing interest.
